# Challenges to the performance of current HIV diagnostic assays and the need for centralized specimen archives: a review of the Consortium for the Evaluation and Performance of HIV Incidence Assays (CEPHIA) repository

**DOI:** 10.12688/gatesopenres.13048.1

**Published:** 2019-07-23

**Authors:** Shelley N. Facente, Michael P. Busch, Eduard Grebe, Christopher D. Pilcher, Alex Welte, Brian Rice, Gary Murphy

**Affiliations:** 1 University of California, San Francisco, San Francisco, CA, 94110, USA; 2 Vitalant Research Institute (formerly Blood Systems Research Institute), San Francisco, CA, 94118, USA; 3 Facente Consulting, Richmond, CA, 94804, USA; 4 The South African DST-NRF Centre of Excellence in Epidemiological Modelling and Analysis (SACEMA), Stellenbosch University, Stellenbosch, South Africa; 5 London School of Hygiene and Tropical Medicine, London, UK; 6 Public Health England, London, UK

**Keywords:** HIV incidence, HIV diagnostics, repository, repositories, archived specimens, HIV

## Abstract

**Background: **New challenges for diagnosis of HIV infection abound, including the impact on key viral and immunological markers of HIV vaccine studies, pre-exposure prophylaxis usage and breakthrough infections, and very early initiation of anti-retroviral treatment. These challenges impact the performance of current diagnostic assays, and require suitable specimens for development and evaluation. In this article we review and describe an archive developed by the Consortium for the Evaluation and Performance of HIV Incidence Assays (CEPHIA), in order to identify the critical features required to create a centralized specimen archive to support these current and future developments.

**Review and Findings:** We review and describe the CEPHIA repository, a large, consolidated repository comprised of over 31,000 highly-selected plasma samples and other body fluid specimen types, with over 50 purposely designed specimen panels distributed to 19 groups since 2012. The CEPHIA repository provided financial return on investment, supported the standardization of HIV incidence assays, and informed guidance and standards set by the World Health Organization and UNAIDS. Unified data from extensively characterized specimens has allowed this resource to support biomarker discovery, assay optimization, and development of new strategies for estimating duration of HIV infection. Critical features of a high-value repository include 1) extensively-characterized samples, 2) high-quality clinical background data, 3) multiple collaborations facilitating ongoing sample replenishment, and 4) sustained history of high-level specimen utilization.

**Conclusion:** With strong governance and leadership, a large consolidated archive of samples from multiple studies provides investigators and assay developers with easy access to diverse samples designed to address challenges associated with HIV diagnosis, helping to enable improvements to HIV diagnostic assays and ultimately elimination of HIV. Its creation and ongoing utilization should compel funders, institutions and researchers to address and improve upon current approaches to sharing specimens.

## Introduction

Over the past three decades, archived HIV samples have allowed commercial and academic researchers to develop and evaluate multiple new HIV assays and HIV diagnostic strategies, including new approaches to measure and monitor HIV prevalence and incidence ^1, 2^. This has allowed us to achieve improved sensitivity and specificity of assays with reduced time to detect infection following HIV acquisition ^3– 8^, attain an increased range of incidence and prognostic biomarkers ^9– 11^, advance techniques for identification of diverse HIV genotypes and recombinant viruses as well as prediction of drug resistance ^12– 18^, and automate and increase throughput of diagnostic platforms ^19, 20^. Through these enhanced strategies we have not only broadened the range of potential specimen types that can be used in diagnostic and incidence testing ^21– 23^, but we have also increased the settings in which testing occurs ^24, 25^ and improved estimates of infection timing ^26, 27^.

Yet despite these advancements, new challenges for diagnosis of HIV infection abound. HIV vaccine studies, increasing pre-exposure prophylaxis (PrEP) usage and PrEP breakthrough infections ^28– 31^, and very early initiation of HIV treatment have improved HIV prevention and care considerably, but require further evaluation of the impact on key viral and immunological markers of HIV, upon which existing diagnostic assays rely ^30, 32– 37^. Vaccine trial participants frequently have anti-HIV positive assay results, known as vaccine-induced seropositivity (VISP), despite being HIV-negative ^34^, and very early use of anti-retroviral treatment (ART) (including with PrEP) can have the opposite effect, leading to non-reactive antibody or ambiguous assay result combinations ^38^. This likely occurs as a result of inhibited antibody production due to lack of sustained antigenic stimulation during a time when viral load is typically spiking ^39, 40^. Rapid reduction in viral load before antibody production is fully underway can lead to lower antibody titers and less time for antibodies to mature; assays may then detect antibodies later than expected, or not at all ^35^. These types of new, increasingly important challenges contribute to potentially inaccurate diagnoses at the individual level and misinterpretations of data on the global stage, including inaccurate national or regional prevalence and incidence estimates, and measurement error against the WHO care cascade indicators and UNAIDS 90-90-90 targets. To reduce data misinterpretation, we require creative approaches to ongoing evaluation and enhancements of diagnostic assays and algorithms, necessitating access to suitable specimens for assay development and evaluation.

Specimen repositories previously built by the Centers for Disease Control and Prevention (CDC), National Institutes of Health (NIH), and others have supported many diagnostic developments to date. However, specimens in those repositories have often had limited distribution and no mechanism for regular replenishment, leading to a resistance of many major funders to allocate money toward sustaining those repositories or investing in additional specimen repository projects. Well-maintained repositories with substantial specimen distribution and replenishment will allow multiple current questions to be answered, and will enable us to anticipate and proactively address future challenges to the diagnosis, monitoring, and evaluation of HIV infection.

## Review and findings

The Consortium for the Evaluation and Performance of HIV Incidence Assays (CEPHIA) was formed in 2011 with funding from the Bill and Melinda Gates Foundation; it was created in specific response to a need for cultivated specimens identified during an international Incidence Assay Critical Path Working Group, convened to review challenges and propose solutions to assay development for use in a surveillance and research context ^41^. CEPHIA was created to support the development of existing and new HIV incidence assays, improve data analysis and interpretation of incidence assay results, and bring consensus to the field. CEPHIA is strengthened by its wide membership (see *Acknowledgements*), including close work with the World Health Organization/UNAIDS Technical Working Group on HIV Incidence Measurement and Data Use ^42^, and a large variety of researchers and funders across the globe (see Table 1 and Table 2). Importantly, the consortium was charged with setting standards and conducting independent evaluations of existing and new incidence assays, in order to remove real or perceived assay developer bias and allow direct, objective comparison of HIV incidence assays. To do this, one of CEPHIA’s primary aims was to establish a repository of samples suited for these purposes.

**Table 1.** Examples of HIV assay development and related research studies supported by CEPHIA.

Study type	Examples
**Focused hypothesis-driven studies**	• How the gut inflammasome and specific HIV antibody subclasses change as HIV infection evolves • How timing of treatment initiation after HIV infection impacts kinetics of HIV reservoir seeding and opportunity for cure
**Non-hypothesis-driven efforts to identify** **novel signatures of recent HIV infection**	• Searches for antibodies reactive to peptoids in a large ‘peptoid shape library’ • Multiplexed assay utilizing viral and antibody markers identified and interpreted through a machine learning algorithm
**CDC and NIH funded projects**	• Examination of the factors in HIV resistance, including mutation, selection, recombination, and drift • Development of a single genomic assay for HIV incidence and transmitted drug resistance mutation screening • Independent evaluation of the Sedia Asanté™ HIV-1 Rapid Recency® Assay, currently in use by PEPFAR at international sites
**Theoretical and toolkit innovations**	• Development of a theoretical framework and web-based tool for consistent time of infection estimation based on subject-level diagnostic testing histories and the properties of diagnostic assays

For this paper, we review the foundation and describe the evolving development of the CEPHIA repository from late 2011 through March 2019, as a potential exemplar of a model for consolidated repository development. We calculate the estimated value of the first stage of the CEPHIA investment (CEPHIA-1, January 2011 through December 2013) by reviewing funding award documentation during that period, and summing the value of all research and programmatic uses of CEPHIA-1 samples between January 2012 and March 2019, then dividing by the total sum of the expenses related to establishment of the CEPHIA-1 repository, as well as maintenance and utilization of the repository during the initial project period. We identify critical features of a high-value repository, for consideration in future efforts to develop such a resource, and identify new types of specimens that could be included in a consolidated repository.

### Review of the CEPHIA Repository

As of March 2019, the CEPHIA repository included 94,654 aliquots of different sample types, collected from 3,383 unique individuals with 13,856 different timepoints. Most of these specimens have well-estimated durations of infection at the time of collection, demographic information, and detailed clinical information about ART history, elite control, and other characteristics.

In addition to performing its own evaluations of 11 existing HIV incidence assays, since 2012 CEPHIA has distributed over 50 purposely designed specimen panels to 19 different research and development groups. Unified data from extensively characterized, highly valuable specimens has allowed this resource to support biomarker discovery, assay optimization, and development of new strategies for estimating duration of HIV infection by interpreting diagnostic histories, as shown in Table 1.

The initial CEPHIA repository was built not by collecting new specimens, but by incorporating historical specimens that otherwise would have been discarded due to lack of storage and maintenance resources. Bringing together smaller, more narrowly-defined repositories added value to specimens that may otherwise have been underutilized or even destroyed, and thus demonstrates the utility of a consolidated repository. However, CEPHIA faced many challenges in the development of its initial repository, as discussed in Box 1. Other groups attempting to build new consolidated repositories will likely face these challenges, making it necessary to determine the return on investment (ROI) required to justify funding for any new repository.

**Box 1. Challenges to developing and maintaining an effective specimen repository**

**Finding specimens**

- Location of specimens with sufficient provenance
- Location of specimens with appropriate volumes
- Methods for obtaining specimens: Collaborate, buy, and/or negotiate
- Replenishment of the archive as specimens are utilized, while maintaining consistency across standard panels available to researchers and assay developers

**Funding**

- Securing of funding for repository development, management and maintenance, including freezer, laboratory technician time to pull and aliquot requested specimens, and data management time
- Demonstration of adequate return on investment, when the value created by disseminated panels is not always readily calculated

**Ethics**

- Marriage of the different ethical requirements of the projects that collected specimens
- Control of specimen usage when supported by commercial companies, who will later sell a product based on evaluations using specimens from the repository

**Ownership and management**

- Creation of appropriate archive management structure
- A priori decisions about the makeup of standard panels
- Determination of who should receive valuable, irreplaceable specimens
- Strategies to appropriately acknowledge the historical projects that collected these specimens

**Data**

- Standardization of the results of evaluations between assays
- Standardization of background data of specimens from many different sources
- Dissemination of results in ways that best benefit the field
- Creation and advertisement of tools to help people with their own analyses

While there are numerous benefits to the field, maintenance of a repository like CEPHIA is costly, and funding to support administrative and operational (i.e. non-research) tasks has been difficult to sustain. Specimens sitting in a freezer waiting to be distributed through a panel are often seen as a drain on resources; yet they can also be seen as a potential supply of invaluable (and sometimes irreplaceable) material, such as in the case of specimens from ART-naïve individuals with longstanding infections, increasingly difficult to come by worldwide.

It is difficult to quantify the value gained from identifying poorly performing assays so they can be removed or limited in the market; improving the understanding, confidence and application of well-performing assays; developing new research concepts and opportunities by adding value to previous studies; or supporting external quality assessment (EQA) programs. Repository value, however, can be measured in several ways, including scientific value (measured through grants awarded, papers published, patents filed, diagnostic improvements made and new assays developed, public health application of methods, and subsequent reduction in infections), regulatory acceptance (through use of the repository to allow FDA, CE, and WHO approval of assays), financial gains (through payments from manufacturers, value of grants awarded, royalties, reduction in ancillary health costs through reduction in burden of disease), and also from a reduction in resources spent on techniques that have been shown to be ineffective. To date, our estimates of expense and funders’ investments into the original plasma-only CEPHIA Repository (also known as “CEPHIA 1”, funded by the Bill and Melinda Gates Foundation, OPP1017716) have had a strong value per dollar invested; when taking into account the value of projects made possible directly or indirectly by CEPHIA 1, we estimate **each dollar invested yielded approximately $6.59 in scientific advancement** (see Table 2).

**Table 2.** CEPHIA value generation.

Item	Expense	Return
**Costs of repository**	**$1,590,978**	
Specimen acquisition	$172,000	
Specimen maintenance	$396,091	
Panel building and shipment	$492,100	
Database management	$315,000	
Oversight	$215,787	
**Projects made possible because of repository**		
NIH awardees		$4,383,760
Gates awardees		$3,000,000
MeSH Consortium incidence applications		$1,300,000
CEPHIA Assay Evaluations		$1,807,223
**TOTAL YIELD**	$10,490,983 / $1,590,978 = **$6.59/$1**	

In addition to the financial yield, there were a number of other impacts of CEPHIA 1, including:

- **Standardization of HIV incidence assays, and rejection of tests that don’t work.** CEPHIA was primarily initiated to conduct independent evaluations of assays in the global market that were theoretically able to detect recency of HIV infection. These evaluations were completed on 11 assays, with formal evaluations (also known as “blue books”) available at the following location: http://www.incidence-estimation.org/page/cephia-assay-evaluations. In addition to these detailed evaluations there were a number of papers published by CEPHIA on the performance and appropriate use of available assays ^43– 45^. These evaluations and related publications have allowed major international donors to confidently use appropriate incidence assays in major surveillance activities and impact assessments, such as the Population HIV Impact Assessments (PHIAs) currently being conducted in 14 PEPFAR-supported countries ^46^.
- **Innovative research to advance the field.** There have been 21 separate large-scale research projects funded by NIH, the Bill and Melinda Gates Foundation, and others, using CEPHIA specimens as a foundation for investigation of assays in various HIV incidence and diagnostic applications. Eleven scientific articles ^2, 11, 26, 27, 43, 45, 47– 51^ highlighting important findings about the use (and misuse) of HIV incidence assays have been published to date with CEPHIA authorship, as a direct result of CEPHIA specimens being made available to researchers upon request.
- **Influence on guidance and standards set by the World Health Organization and UNAIDS.** Through participation on the WHO/UNAIDS Technical Working Group on HIV Incidence Measurement and Data Use, CEPHIA has had direct and ongoing influence over the information discussed at Technical Working Group meetings and subsequently shared through various guidance documents and technical updates, available at https://www.who.int/hiv/pub/surveillance/en/; as one example, this influence led to a 2015 Technical Update recommending that incidence assays always be used in conjunction with viral load to appropriately calculate incidence at the population level ^52^.
- **Research informed by CEPHIA analyses and lessons learned.** In addition to specific publications, many areas of research and development have been largely informed by CEPHIA’s work, including HIV cure research, antibody profiling, and infection dating. As one concrete example, to support analyses within CEPHIA it was critical to standardize estimated times since infection for each specimen timepoint. This was originally done using the conventionally-understood Fiebig staging method ^53^. However, this was quickly found to be limiting as many specimens came from people who entered the source cohort after reaching Fiebig stage V, when antibodies had matured and the acute infection period had passed, yielding little-to-no information about infection timing. To address this limitation, CEPHIA developed a new method of infection dating now gaining traction ^26, 27^; the major benefit of the EDDI method is that it allows for a point estimate and associated credibility interval for date of first detectable infection of any person who has at least one positive and one prior negative HIV test of any kind. With the EDDI method, the tests do not have to be run on the same day and do not have to be run during the acute phase of infection in order to generate an estimated date on which a viral load assay with a 1 copy/mL limit of detection would have a 50% chance of detecting the infection (date of first “detectable infection”). The EDDI method is flexible and a major advance over the Fiebig method given the latter’s increasing limitations in this new era of HIV assays.

### Critical features of a high-value repository

**Figure 1.**
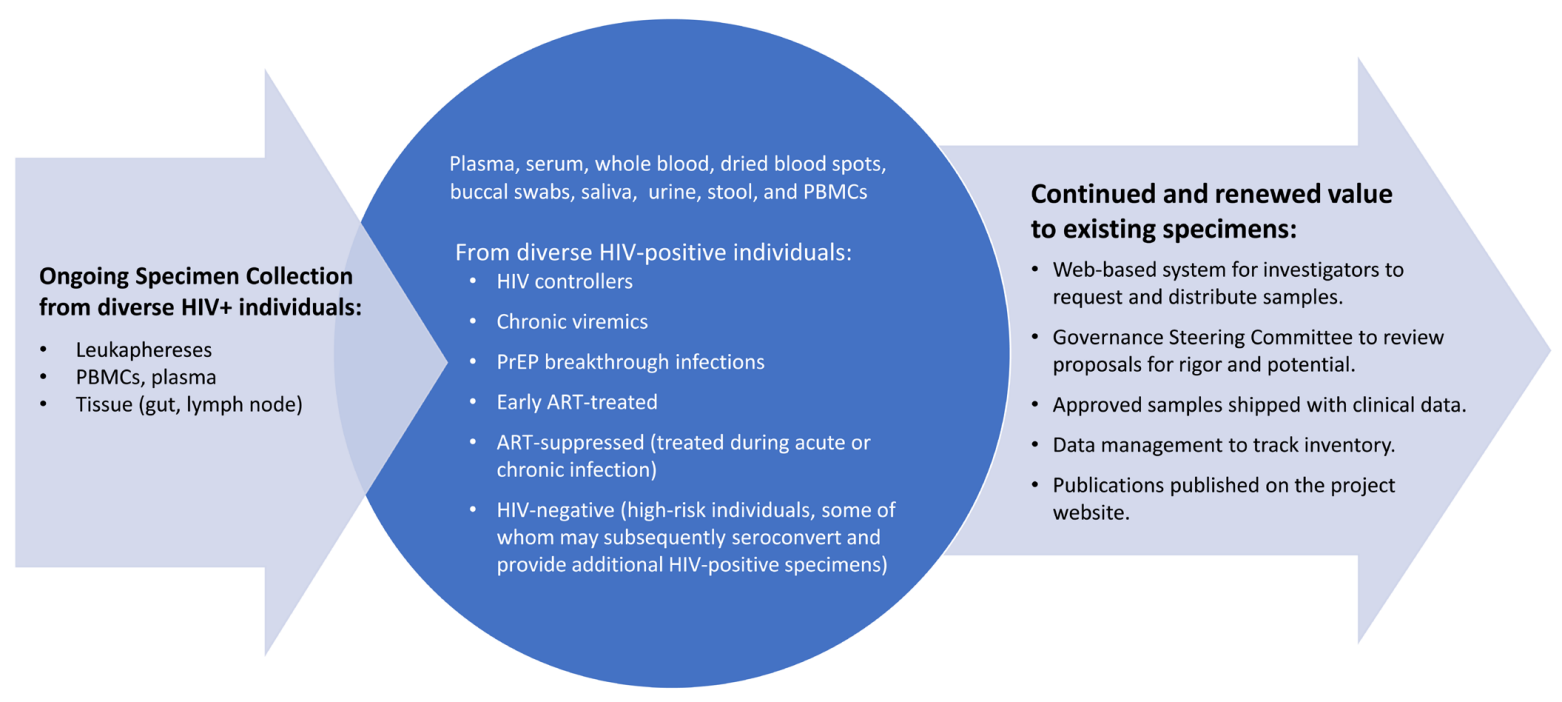
Consolidated repository concept.

We identified the following as critical features of a high-value repository: 1) extensively-characterized HIV-positive samples, including serial specimens from people with acute infection, seroconverters, and treated subjects, enabling diagnostic, pathogenesis, cure, and co-morbidity studies, 2) high-quality clinical background data, 3) multiple collaborations facilitating ongoing specimen collection and replenishment, and 4) sustained records of high-level utilization and specimen turnover, with thousands of samples shared annually. In Figure 1, we describe the features of a large consolidated repository that meets these “high-value” criteria on a broad scale with centralized management.

Given the changing context of HIV diagnostics in 2019, we identified the need for new types of specimens that should be available through a consolidated repository. In order to sufficiently support the development of assays that will meet today’s diagnostic challenges, we need a repository with the infrastructure to support rapid receipt, management, and dissemination of panels with the following types of specimens:

1. Large-volume, extensively-characterized HIV-positive samples, including serial specimens from seroconverters and patients with particularly early ART initiation;
2. Baseline samples from individuals starting PrEP and serial samples collected while on and after stopping PrEP, including from documented and potential PrEP-breakthrough infections;
3. Chronic viremics (i.e. not virally suppressed even with longstanding infection)
4. Elite controllers (i.e. continuously virally suppressed despite not having been treated)

All specimens must have high-quality clinical background data on the patients, to ensure their utility for diagnostic, pathogenesis, cure, and co-morbidity studies. This will require multiple collaborations to facilitate specimen collection and replenishment; this is no small task considering the legal, ethical, and logistical implications of sharing large-volume specimens across the globe, for reasons other than their original intention. An effective repository should have thousands of samples shared annually, requiring extensive data management to track shipments, specimen usage, panel outputs, and high-level specimen turnover and replacement. A repository system with the capacity to independently evaluate assays that have performed well on blinded panels run in developers’ laboratories (as CEPHIA has) also requires collaboration with advanced statisticians who can perform high-quality data analysis and routinely share information; our experience has shown that simply disseminating evaluation results is not sufficient, and hands-on technical assistance strategies are required in order to support clinicians and researchers in understanding what the results really mean.

## Discussion

Despite having a reputation as expensive white elephants, our review highlights that specimen repositories can play an invaluable role in the development, optimization, and validation of diagnostic assays. The CEPHIA repository demonstrates how a targeted approach can identify and capture a wealth of specimens and associated information that already exists, and *gain* value by being drawn together for a different purpose. Developing the archive required overcoming a number of significant challenges, and despite its demonstrated value given the financial investment, lack of consistent funding threatens the existence of the CEPHIA repository and the collaborative team that supports its use. To successfully develop and sustain a consolidated repository model, we must think in new ways about our scientific work as part of a collective whole. To this end, through this review we have identified critical features of a high-value repository, leading to the following proposals:

1. **Funders of studies should be more explicit in their guidance to awardees about requirements to release specimens utilized for the study as soon as any primary or secondary aims of the initial study are complete**, and this should be completed within a defined timeframe. ‘Ownership’ of specimens collected through studies is a significant hurdle to overcome in the development of a consolidated repository. Understandably, individuals typically do not want to release specimens held in their archives without guarantees that the work performed on them is going to be valuable, and institutions worry about yielding control of specimens under their purview. Indeed, specimens in a consolidated repository may be used in ways that diverge from the expertise of the researchers who were instrumental in their collection, often leading to further reluctance to collaborate. Funders should also look across the repertoire of studies they fund, to help identify where specimens may be available and how specimens collected from one study may be beneficial to others.
2. **In developing ethical approval for studies, more emphasis should be placed on ensuring that specimens collected can be used for other, perhaps currently unknown, purposes.** This may mean setting a period of time for the study after which anonymization of specimens is routinely conducted so they may be broadly shared. It will also likely require that further explanation of the potential uses of specimens be given to study participants at the time of consent.
3. **Journals should come to some general consensus about the best mechanisms for acknowledging researchers who contributed specimens that were later used for a secondary study.** In a large repository with rapid turnover of specimens, inclusion of all researchers contributing specimens into the authorship list of any subsequent papers is likely impractical. Yet especially in academia, where a publication record is typically a major factor in continued employment and promotion, rights to authorship is critical currency when determining whether specimens should be shared with a repository system for future use. Recognition of the hard work that went into specimen acquisition and initial maintenance – as well as incentivization for collegial relationships between researchers – would be an important way to ensure successful construction of a consolidated repository.
4. **Major funders should ensure that studies collect as much ancillary data around the work they do as possible without infringing on the rights of study participants.** While most researchers work to minimize and streamline data collection to improve participant enrollment rates (i.e. not requiring participants to disclose full medical histories), there are many simple factors of study protocol or process that could help with the effective use of specimens for subsequent analyses. One clear example of this is the type of assay used to confirm an infection, which due to different sensitivities and specificities may, over time, affect how an assay result may be interpreted or how a specimen may be categorized.
5. **Leadership of a consolidated repository should be supported by the endorsement of major funders and normative agencies.** With international, influential commitments to the development and use of a major repository to address upcoming challenges to HIV diagnostics, the ease of securing appropriate specimens will dramatically increase. The repository should be open internationally, with its use based on scientific need, with clear systems and guidelines for specimen access. Further, management of the repository should not just include physically obtaining, storing, and shipping specimens, but should also involve skilled support for data analysis, to help guide users in proper application of the specimens and their associated information. Standards should be set for commercial use of specimens, such that for-profit development is allowed with an appropriate exchange of funding that helps the repository be sustained for the future.
6. **Repository owners should attempt to develop an ROI methodology.** To assist with sustainable funding for useful consolidated repositories, a mechanism to fairly track investments, expenses, and returns is imperative. Such a system will help potential funders see the benefits of their investments in these projects, and should also include metrics to determine whether a repository should continue or whether the ROI has fallen to a point such that future funding should cease and specimens should be disseminated for storage in other more productive settings.

In summary, with strong governance and leadership, a consolidated repository provides academic investigators and commercial assay developers with easy access to diverse specimens through a large consolidated archive of specimens from multiple studies, helping to advance improvements to HIV diagnostic assays and ultimately elimination of HIV. An international commitment to the development and/or maintenance of this type of resource is imperative, for the field of HIV diagnostics and incidence measurement to continue to move forward and face new challenges.

## Data availability

No data is associated with this article.
