# Supplementary material for: Challenges to the performance of current HIV diagnostic assays and the need for centralized specimen archives: a review of the Consortium for the Evaluation and Performance of HIV Incidence Assays (CEPHIA) repository
Source: Gates Open Res. 2019 Jul 23;3:1511. [Version 1] doi: 10.12688/gatesopenres.13048.1 (PMC6706958; doi:10.12688/gatesopenres.13048.1)
Supplement: Peer review history

# Peer review: Reviewer response for version 1

**DOI:** 10.21956/gatesopenres.14170.r27669

**Published:** 2019-08-30

**Authors:** Elliot P. Cowan

**Affiliations:** 1 Partners in Diagnostics, LLC, Rockville, MD, USA

This Open Letter describes the creation of a specimen repository to advance the development of HIV diagnostic assays. It makes its points clearly, identifying not only the value of the CEPHIA repository but also the challenges encountered in assembling it. I see the assembly of the repository a remarkable achievement that should be a model for the generation of other repositories for other infectious agents and diseases. I found the proposals at the end of the Open Letter to be especially provocative (in its most positive sense), presenting clear paths forward that will undoubtedly go a long way in getting the most out of the repository. Some comments to consider:

 The Letter correctly sees value in the repository for, “regulatory acceptance (through use of the repository to allow FDA, CE, and WHO approval of assays).” It will be important to understand from those bodies what they expect for that acceptance. This especially includes ethical issues (which the authors recognize as important to address).

 The repository will be an important tool in product development by IVD manufacturers. How do the authors, or do they, envision making repository samples available to product developers?

 Understandably, the CEPHIA repository took several years to assemble and continues to be a work in progress. I would like the authors to comment on how they can apply their experience to assembling a repository when there is a public health emergency, in which assays are desperately needed and critical specimens for test development and validation are extremely difficult to source.

I confirm that I have read this submission and believe that I have an appropriate level of expertise to confirm that it is of an acceptable scientific standard.

# Peer review: Reviewer response for version 1

**DOI:** 10.21956/gatesopenres.14170.r27584

**Published:** 2019-08-22

**Authors:** Bernard M. Branson

**Affiliations:** 1 Scientific Affairs LLC, Atlanta, GA, USA

This open letter describes the CEPHIA repository and its role in development of evaluation of HIV incidence assays, and proposes it serve as a “potential exemplar for consolidated repository development.” The authors note (Review and Findings) that “CEPHIA was created to support the development of existing and new HIV incidence assays, improve data analysis and interpretation of incidence assay results, and bring consensus to the field,” and note (Discussion) that “lack of consistent funding threatens the existence of the CEPHIA repository and the collaborative team that supports its use.” I have significant reservations about this letter from my eminent colleagues, and I provide extensive comment because of my respect for the hard work these colleagues have done.

 The prospect of using a serologic assay to directly measure (or estimate) HIV incidence from cross-sectional specimen collection first showed promise using a somewhat primitive modified ELISA in 1998 (Janssen *et al.*, 1998 ^2^). Subsequent assays were developed to address the shortcomings identified with each later iteration of assays (Busch *et al.*, 2010 ^3^), but, as specified in several of the authors’ citations, no single assay method can precisely estimate incidence; WHO guidance stipulates that assay-based estimation is not absolute (World Health Organization, 2018 ^4^); and other studies by these same authors demonstrate that assay-based incidence estimates with the same specimens differ depending on the assay, specimen type, and context (Schlusser *et al.*, 2017 ^5^ and Sempa *et al.*, 2019 ^6^). These cause me concern about the implications of the recent proposed deployment of the rapid POC “recency” assay (authors’ citation 46) reminiscent of the premature distribution of the BED assay. https://apps.who.int/iris/bitstream/handle/10665/233008/WER8104_40-40.PDF?sequence=1&isAllowed=y.

 At CEPHIA’s inception, acquisition of specimens collected from other studies was anticipated as a shortcut, quicker and less expensive than a purposefully designed prospective specimen collection from an observational cohort. However, the best sources for CEPHIA specimens were other cohorts (e.g., Zvitambo, CHAVI) (Gonese *et al.*, 2019 ^7^ and Seaton *et al.*, 2017 ^8^) with limited specimen volumes for which there are other competing research interests. The CEPHIA panel suffers from several shortcomings in that many specimen sets are based on estimated (not observed) dates of infection, and the panel constituents do not allow direct comparison of assay-based incidence results to observed incidence. Finally, the CEPHIA panel will also not help address the pressing new challenges for diagnosis in persons taking PrEP, PEP, or early ART, and will not allow for examination of new molecular methods or POC specimen types, for which better specimen sources are available (i.e., SEARCH studies, ClinicalTrials.gov NCT00796146 and NCT00796263, underway since 2008) (Ananworanich *et al.*, 2013 ^9^ and de Souza *et al.*, 2016 ^10^).

 After 20 years’ effort, serologic incidence estimation has yet to achieve proof of concept. All survey results require extensive mathematical manipulation, and reinterpretations are frequent. All assay methods evaluated to date extensively misclassify persons on ART, which inflates incidence estimates.  CDC’s domestic surveillance has abandoned serologic incidence assays in favor of a CD4 depletion model (Hall *et al.*, 2017 ^11^).

 The authors do not indicate why they believe collection of specimens and maintenance of a repository should not be a government responsibility (e.g., DoD HIV specimen repository (Perdue *et al.*, 2015 ^12^), Lyme repository (Molins *et al.*, 2014 ^13^), CHAVI, ACTG, HPTN, and MACS HIV specimen repositories]), nor why a prospective plan is not designed and implemented, such as the NHLBI national blueprint for 21st century data and specimen collection and observational cohort studies (Konkle and Recht, 2019 ^14^).

 Finally, the authors’ estimates of the repository appear to neglect the fixed costs associated with development, maintenance, and monitoring of a repository (Baird and Gunter, 2016 ^15^), and I am reluctant to equate grants received (including, I expect, some by these authors) as ROI. If ROI justified incidence assays, industry would underwrite the specimen repositories.

I confirm that I have read this submission and believe that I have an appropriate level of expertise to confirm that it is of an acceptable scientific standard, however I have significant reservations, as outlined above.

## References

[1] : More and better information to tackle HIV epidemics: towards improved HIV incidence assays. *PLoS Med*.2011;8(6) : 10.1371/journal.pmed.1001045 e1001045 10.1371/journal.pmed.1001045 21731474PMC3114871

[2] : New testing strategy to detect early HIV-1 infection for use in incidence estimates and for clinical and prevention purposes. *JAMA*.1998;280(1) : 10.1001/jama.280.1.42 42-8 10.1001/jama.280.1.42 9660362

[3] : Beyond detuning: 10 years of progress and new challenges in the development and application of assays for HIV incidence estimation. *AIDS*.2010;24(18) : 10.1097/QAD.0b013e32833f1142 2763-71 10.1097/QAD.0b013e32833f1142 20975514

[4] : Meeting Report: WHO Working Group on HIV Incidence Measurement and Data Use.2018; Reference source

[5] : Comparison of cross-sectional HIV incidence assay results from dried blood spots and plasma. *PLoS One*.2017;12(2) : 10.1371/journal.pone.0172283 e0172283 10.1371/journal.pone.0172283 28231277PMC5322916

[6] : Performance comparison of the Maxim and Sedia Limiting Antigen Avidity assays for HIV incidence surveillance. *PLoS One*.2019;14(7) : 10.1371/journal.pone.0220345 e0220345 10.1371/journal.pone.0220345 31348809PMC6660077

[7] : Evaluation of the Performance of Three Biomarker Assays for Recent HIV Infection Using a Well-Characterized HIV-1 Subtype C Incidence Cohort. *AIDS Res Hum Retroviruses*.2019;35(7) : 10.1089/AID.2019.0033 615-627 10.1089/AID.2019.0033 30938164PMC10719552

[8] : Computational analysis of antibody dynamics identifies recent HIV-1 infection. *JCI Insight*.2017;2(24) : 10.1172/jci.insight.94355 10.1172/jci.insight.94355 PMC575230029263306

[9] : A novel acute HIV infection staging system based on 4th generation immunoassay. *Retrovirology*.2013;10: 10.1186/1742-4690-10-56 56 10.1186/1742-4690-10-56 23718762PMC3669623

[10] : Initiation of Antiretroviral Therapy During Acute HIV-1 Infection Leads to a High Rate of Nonreactive HIV Serology. *Clinical Infectious Diseases*.2016;63(4) : 10.1093/cid/ciw365 555-561 10.1093/cid/ciw365 27317797

[11] : HIV Trends in the United States: Diagnoses and Estimated Incidence. *JMIR Public Health Surveill*.2017;3(1) : 10.2196/publichealth.7051 e8 10.2196/publichealth.7051 28159730PMC5315764

[12] : A Brief Description of the Operation of the DoD Serum Repository. *Mil Med*.2015;180(10 Suppl) : 10.7205/MILMED-D-14-00739 10-2 10.7205/MILMED-D-14-00739 26444888

[13] : Collection and characterization of samples for establishment of a serum repository for lyme disease diagnostic test development and evaluation. *J Clin Microbiol*.2014;52(10) : 10.1128/JCM.01409-14 3755-62 10.1128/JCM.01409-14 25122862PMC4187768

[14] : The national blueprint for 21st century data and specimen collection and observational cohort studies: NHLBI State of the Science Workshop on factor VIII inhibitors. *Haemophilia*.2019;25(4) : 10.1111/hae.13772 590-594 10.1111/hae.13772 31329362

[15] : Repository Planning, Design, and Engineering: Part II-Equipment and Costing. *Biopreserv Biobank*.2016;14(4) : 10.1089/bio.2015.0085 338-49 10.1089/bio.2015.0085 26886768

# Peer review: Reviewer response for version 1

**DOI:** 10.21956/gatesopenres.14170.r27583

**Published:** 2019-08-21

**Authors:** Niel T. Constantine

**Affiliations:** 1 Institute of Human Virology and Department of Pathology, University of Maryland School of Medicine, Baltimore, MD, USA

**General**: The paper is well-written and of importance for scientists in the diagnostics arena. The effort is noteworthy, and the challenges of such a repository are evident. The authors are experts in the field. A few comments for improvement are indicated below.

**Abstract:**

1. The authors indicate 31,000 plasma specimens, but in the Review of the CEPHIA Repository section, they state 3,383 individuals with 13,856 different time points. The repository number and type of specimens are a little unclear, and perhaps the number and characteristics of the population of samples could be further clarified in one section.
2. The authors state “…other body fluids…”. This is not described in subsequent sections and should be further explained.
3. The abstract mentions “support biomarker discovery”. This should be further explained in a subsequent section.

**Table 2: **

- For the cost of the repository, the expenses could include more detail. For example, the database management is $315,000: what exactly does this involve? Also, specimen maintenance is $396,091: what does this entail? Are these costs per year?

**Box 1: **

- Ethics: what is meant by ethical requirements?
- Ownership: how is it determined who should receive valuable specimens?

**Page 6:**

- EDDI method: not well defined or explained.
- Critical Features: the authors do not indicate the number of serial samples or number of seroconversion panels. Perhaps this should be stated with the total number of samples and time points (as stated in the Abstract). 

**Figure 1:**

- The authors mention whole blood and PBMCs. How would these be processed and stored for use, and how would they be used by users?

**Discussion:**

- “expensive white elephant” - what does this mean? I would suggest not to use this phrase; many readers would not understand what this means. Also, how would it be determined if projects are “going to be valuable”?

**Data Availability:**

- Should be corrected to: “No data are associated…”

**Number 2 question above**:

- The authors could add statements on the opinions of others as to the value for the repository and any differing views or discussions by others.

I confirm that I have read this submission and believe that I have an appropriate level of expertise to confirm that it is of an acceptable scientific standard.

# Peer review: Reviewer response for version 1

**DOI:** 10.21956/gatesopenres.14170.r27582

**Published:** 2019-08-08

**Authors:** Gavin A. Cloherty

**Affiliations:** 1 Infectious Disease Research, Diagnostics Division, Abbott Laboratories, Chicago, IL, USA

The letter clearly describes the history of the CEPHIA repository. 

 This letter is valuable in that it describes the hurdles faced by any organization trying to establish a sample repository for research purposes and makes recommendations for how those hurdles might be overcome.

 The authors do a very good job describing the value (often unrecognized or difficult to quantify) of such a repository. They also accurately describe the challenges facing diagnostic manufacturers, diagnostic laboratories and physicians caused by novel approaches to HIV care.

I confirm that I have read this submission and believe that I have an appropriate level of expertise to confirm that it is of an acceptable scientific standard.
